# Evolution of knee osteoarthritis biomechanics research, 2005–2025: bibliometric mapping and machine-learning-assisted topic modeling

**DOI:** 10.3389/fmed.2026.1968099

**Published:** 2026-10-26

**Authors:** Fei Lu, Lu Liu, Peng Yang, Hang Gao, Hang Song, Guoquan Wang, Zongbao Wang

**Affiliations:** 1 School of Acupuncture and Massage, Anhui University of Chinese Medicine, Hefei, Anhui, China; 2 College of Integrated Chinese and Western Medicine, Anhui University of Chinese Medicine, Hefei, Anhui, China; 3 Functional Activity and Resource Utilization of Edible and Medicinal Fungi Anhui Provincial Joint Key Laboratory, Anhui University of Chinese Medicine, Hefei, Anhui, China; 4 Anhui Provincial Key Laboratory of Tumor Evolution and Intelligent Diagnosis and Treatment, Bengbu, Anhui, China

**Keywords:** biomechanics, knee osteoarthritis, non-negative matrix factorization, research trends, topic modeling

## Abstract

**Background:**

Knee osteoarthritis (KOA) involves interactions among whole-joint loading, tissue mechanics, and cellular responses. This study mapped the structure and temporal evolution of KOA biomechanics research using bibliometric analysis and machine-learning-assisted topic modeling.

**Methods:**

English-language articles and reviews published from 2005 to 2025 were retrieved from the Web of Science Core Collection and Scopus, merged, deduplicated, and screened. The final corpus comprised 3110 publications. Publication patterns, collaboration networks, citation structures, and keyword themes were evaluated, and non-negative matrix factorization was applied to bibliographic text to identify latent topics and their temporal distribution.

**Results:**

Annual output increased from 37 publications in 2005 to 321 in 2025. The United States led in publication output and total citations, while the University of Melbourne was the most productive institution. Keyword analysis identified five major domains spanning mechanobiology, gait and neuromuscular control, conservative load-modifying interventions, alignment correction and reconstruction, and meniscal and ligament mechanics. The selected model comprised 19 topics. Gait and lower-limb kinematics had the largest overall share. The greatest increases in mean annual topic share from 2016-2020 to 2021-2025 involved stem cells and regenerative cartilage repair, chondrocyte mechanotransduction, and meniscal tears, extrusion, and root repair. Recent changes in topic share were strongly correlated between hard and normalized soft assignments (Spearman's *ρ* = 0.926).

**Conclusion:**

Overall, publication output increased, and topic-share patterns indicated greater relative representation of mechanobiology, regenerative repair, meniscal preservation, and alignment correction, while gait and loading research remained prominent. These findings provide a descriptive map of the field's development and changing research priorities.

## 1 Introduction

Knee osteoarthritis (KOA) is a common chronic musculoskeletal disorder characterized by pain, stiffness, impaired mobility, muscle weakness, and progressive loss of joint function (1, 2). It is a major cause of disability among middle-aged and older adults and imposes a substantial burden on patients, families, and healthcare systems (3, 4). Population aging and the rising prevalence of obesity are expected to increase the number of people affected by KOA (3, 5). A clearer understanding of its pathogenesis is therefore needed to improve prevention and management, delay disease progression, and optimize treatment.

KOA is a multifactorial disorder associated with aging, sex, obesity, previous knee injury, lower-limb malalignment, muscle dysfunction, inflammation, metabolic abnormalities, and genetic susceptibility (6–8). Within this complex pathological framework, biomechanics links systemic and anatomical risk factors to local joint degeneration (9, 10). Body mass, lower-limb alignment, gait patterns, muscle strength, neuromuscular control, joint instability, and previous injury affect knee loading at the whole-body and joint levels (7, 8, 10, 11). These factors influence the magnitude, distribution, direction, and repetition of loading. Altered loading may change tibiofemoral and patellofemoral contact mechanics, redistribute loads between joint compartments, and increase mechanical stress in vulnerable regions (12, 13). The knee adduction moment (KAM), knee flexion moment (KFM), joint contact force, loading rate, and dynamic alignment are therefore widely studied indicators of the knee's mechanical environment. These factors may help explain the spatial heterogeneity of structural degeneration. They may also explain why patients with comparable structural disease have different movement patterns, symptoms, and rates of progression (14–16).

The knee's mechanical environment reflects external loads, movement patterns, joint alignment, stability, and muscle function (17). Together, these factors determine how loads are transmitted through articular cartilage, the menisci, subchondral bone, and other periarticular structures (18). At the tissue and cellular levels, these structures respond continuously to mechanical stimuli. Excessive, insufficient, or abnormally distributed loads can disrupt extracellular matrix metabolism and cellular mechanotransduction, promoting interactions between inflammatory and reparative responses (19, 20). Clarifying how whole-joint biomechanics and local tissue loading relate to KOA onset and progression is therefore a central research objective (21). Motion capture, electromyography, medical imaging, wearable sensors, musculoskeletal modeling, and finite-element analysis are increasingly combined to quantify movement and estimate internal joint forces, tissue stress, and tissue strain (22, 23). Such multidimensional research also supports the development of interventions targeting mechanical factors (24). Some interventions alter joint-loading parameters or improve symptoms, but responses vary substantially among individuals. Evidence that modifying the mechanical environment can sustainably delay structural progression remains limited.

Conventional keyword analysis identifies explicitly named research concepts and their relationships, but depends on author terminology, indexing practices, and keyword-standardization procedures (25). Non-negative matrix factorization (NMF) provides a complementary document-level perspective by identifying weighted thematic components in bibliographic text, supporting a more detailed characterization of research topics (26). Aggregating document-level topic assignments by publication year also enables the examination of changes in relative topic prevalence over time (27). In KOA biomechanics, this approach can help distinguish related research strands, such as chondrocyte mechanotransduction and regenerative cartilage repair, and examine whether they follow different temporal trajectories.

This study analyzed English-language articles and reviews on KOA biomechanics published between 2005 and 2025 and indexed in the Web of Science Core Collection and Scopus. We integrated conventional bibliometric methods with machine-learning-assisted topic modeling to describe the field's development, current knowledge structure, and changing research priorities.

## 2 Materials and methods

### 2.1 Data sources and search strategies

The Web of Science Core Collection (WoSCC) and Scopus were searched on July 26, 2026, for English-language articles and reviews published from January 1, 2005, through December 31, 2025, covering the entire 2025 calendar year. English-language records were selected to support consistent term standardization and a common vocabulary for NMF modeling based on term frequency–inverse document frequency (TF–IDF). Articles and reviews were included to capture original research and research syntheses while limiting heterogeneity associated with additional publication formats. Searches used the Topic (TS) field in WoSCC and TITLE-ABS-KEY in Scopus. In both databases, KOA-related terms, including “knee osteoarthri*,” “KOA,” and “knee OA,” were combined using AND with biomechanics and mechanical-loading terms, including “biomechanic*,” “mechanical load,” “mechanical stress*,” and “joint loading.” Database-specific syntax was applied, and the complete search strategies are provided in Supplementary Table S1. The study selection and analysis workflow is shown in Figure 1.

**Figure 1:**
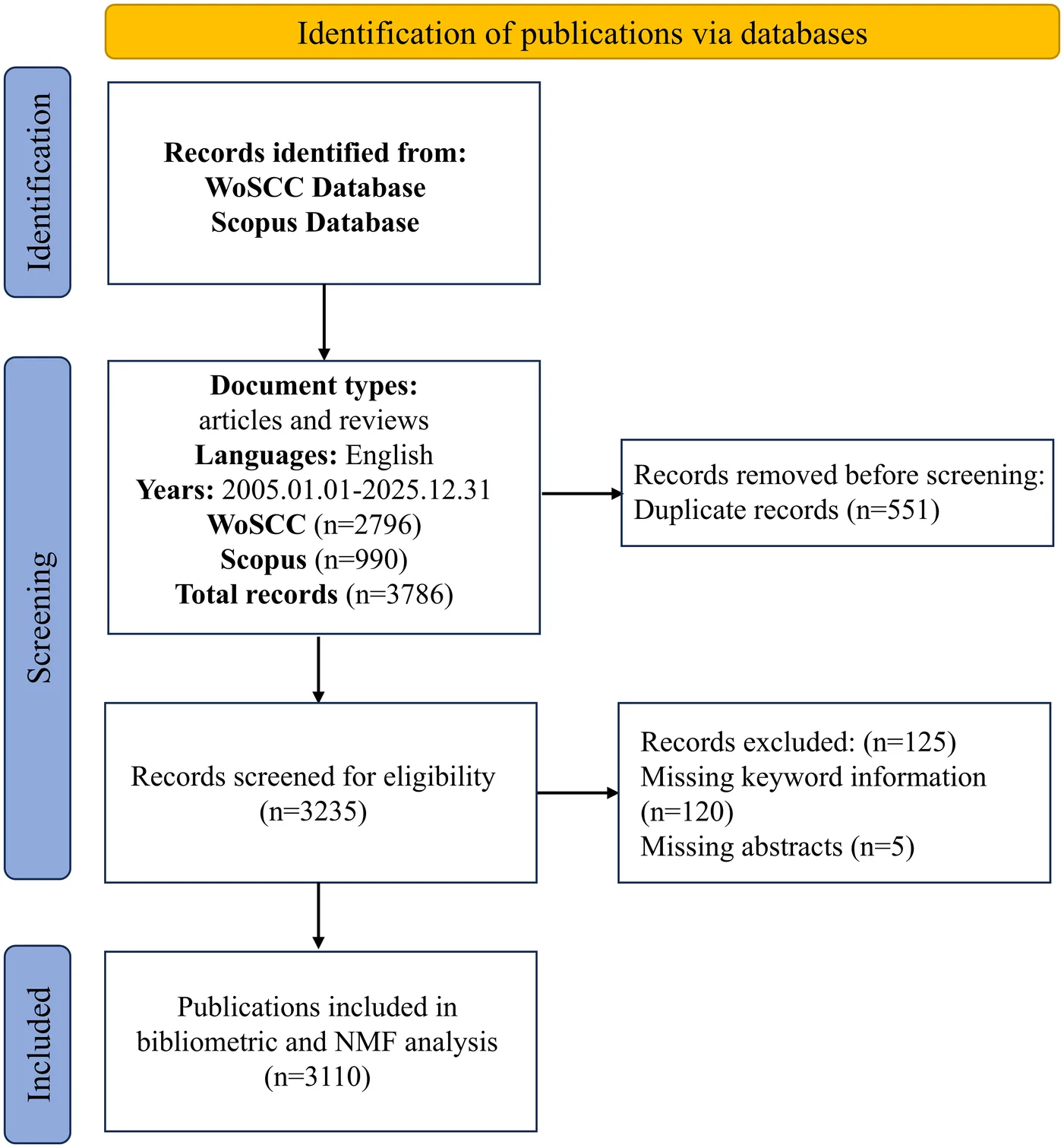
Study selection and analysis workflow.

### 2.2 Study selection and data screening

Records were exported separately from WoSCC (*n* = 2,796) and Scopus (*n* = 990). Scopus records were converted in Python into a WoS-compatible plain-text format by mapping the corresponding bibliographic fields before merging. Of the 3,786 retrieved records, 551 duplicates were identified primarily by DOI and title and removed, leaving 3,235 records for eligibility screening. Two investigators independently screened the titles, abstracts, and keyword fields of the retrieved records to assess their relevance to KOA biomechanics and compliance with the eligibility criteria. A total of 125 records were excluded because of missing keyword information (*n* = 120) or missing abstracts (*n* = 5). Disagreements were resolved through discussion or, when necessary, adjudication by a third senior investigator. The final corpus comprised 3,110 unique publications and was used for both bibliometric analysis and NMF-based topic modeling.

### 2.3 Data preprocessing and descriptive bibliometric analysis

Eligible records were merged into a unified WoS-compatible dataset and imported into R (version 4.4.3). Analyses were conducted in RStudio using bibliometrix (version 4.5.1). Author names, affiliations, countries or regions, and keywords were standardized. Keyword preprocessing included case normalization, singular–plural consolidation, abbreviation expansion, and synonym merging. Two investigators independently reviewed the thesaurus, and a third investigator resolved disagreements. Indicators included annual output and total and average citations. We also assessed publication and citation contributions by countries or regions, institutions, authors, and journals. Bibliometrix was used to calculate single-country publications (SCPs), multiple-country publications (MCPs), and MCP ratios. It was also used to assess author productivity using Lotka's law, generate author publication timelines, and identify core journals using Bradford's law. The journal publication-output figure was generated with bibliometrix and optimized in RStudio without altering the underlying data. Annual publication output was summarized descriptively using an exponential curve fitted in Microsoft Office Excel 2019. The independent variable was the year index, and the coefficient of determination (R^2^) was reported only as a goodness-of-fit statistic. The fitted curve was not used to infer an underlying exponential growth process, identify change points, or define discrete developmental stages. Using the final corpus of 3,110 publications, we cross-tabulated the 19 NMF topics by country/region using the original document-level hard assignments described in Section 2.5. Country attribution followed the same procedure as the main country-level analysis. For the ten most productive countries/regions, we reported topic-specific publication counts and within-country percentages, calculated using each country/region's total included publications as the denominator.

### 2.4 Science mapping, citation analysis, and temporal keyword analysis

CiteSpace (version 6.4.R1) was used to analyze country or region co-occurrence, cited-reference co-citation, and citation bursts from 2005 to 2025. CiteSpace analyses used one-year time slices and g-index selection (*k* = 25), with a link-retaining factor of 2.5, L/N = 10, look-back years = 5, *e* = 1.0, and Pathfinder pruning. In the country or region network, node size represented publication output, links represented collaboration, and link thickness represented collaboration strength. In the cited-reference network, node size represented co-citation frequency, and links represented co-citation relationships. Citation bursts identified references with rapid increases in citations during specific periods. VOSviewer (version 1.6.20) was used to construct the author-keyword co-occurrence network. After thesaurus-based standardization, keywords occurring at least 20 times were included. Full counting and association-strength normalization were applied. Node size represented occurrence frequency, link thickness represented pairwise co-occurrence strength, and node color represented cluster membership. The final author-keyword network comprised five color-coded clusters. Historical keyword evolution was assessed using annual keyword data generated with bibliometrix. For each keyword, the horizontal line showed the interquartile range of publication years, the point showed the median year, and point size showed cumulative frequency. The timeline figure was optimized in RStudio without altering the data or results.

### 2.5 Machine-learning-assisted topic modeling and retrospective temporal analysis

#### 2.5.1 Machine-learning-assisted topic modeling

Available titles, abstracts, and standardized author keywords were concatenated, with Keywords Plus additionally included for WoSCC records. Lowercased word unigrams and bigrams were transformed using TF–IDF with min_df = 5, max_df = 0.85, max_features = 6,000, sublinear term-frequency scaling, smoothed inverse document frequency, and L2 normalization. Stop words comprised 318 scikit-learn English stop words and 82 custom terms, yielding 400 unique terms. Detailed preprocessing and model settings are provided in Supplementary Table S2, and the complete stop-word list is provided in Supplementary Table S3. Reference NMF models (*K* = 8–20) used nndsvda initialization, coordinate descent, Frobenius loss, max_iter = 1,000, and random_state = 42. Models were evaluated for reconstruction error, coherence (mean normalized pointwise mutual information), distinctiveness, prevalence, and reproducibility. *K* = 19 was selected through sequential eligibility, coherence, and error-improvement criteria (Supplementary Tables S4–S6). Topic labels were assigned based on high-weight terms and 10 representative publications per topic. Publications were ranked within each hard-assigned topic by document-normalized topic weight, with raw weight resolving ties. Two researchers independently reviewed the proposed topic labels, and disagreements were adjudicated by a third independent researcher.

#### 2.5.2 Topic reproducibility and retrospective temporal analysis

For each candidate K, reproducibility was assessed through 30 full-corpus refits with random initialization and 50 year-stratified refits using 80% of documents within each year, sampled without replacement. Subsample refits used nndsvda initialization. The TF–IDF vocabulary and weights were held fixed, and subsampling was performed on rows of the full-corpus TF–IDF matrix. Refit topic–term vectors were aligned with the reference solution using cosine similarity and Hungarian one-to-one matching. Model-level reproducibility statistics pooled the matched similarities across topics and refits for each K, with the results reported in Supplementary Table S5. P10 denotes the 10th percentile of these similarities, rather than a confidence bound. Topic-level P10 values were evaluated separately, and topics with initialization P10 < 0.70 or subsample P10 < 0.50 were interpreted cautiously. Temporal patterns for these topics were treated as exploratory and were not used as standalone evidence of emerging or declining research priorities. Annual topic shares were calculated primarily from each document's highest-weight topic. Recent change was defined as the difference between mean annual shares in 2021–2025 and 2016–2020. Long-term direction was summarized using linear slopes for 2005–2025. Normalized soft topic weights were used in a sensitivity analysis. All temporal analyses were retrospective and descriptive, with no statistical significance testing.

To assess sensitivity to database heterogeneity, TF–IDF and NMF were refitted on the 2,791 WoSCC-indexed records using original WoSCC text fields, the same preprocessing settings, and *K* = 19. Topics were aligned with the combined-corpus model using cosine similarity and one-to-one matching, and recent changes in topic shares were compared.

## 3 Results

### 3.1 Analysis of publications

The analysis included 3,110 publications, and annual publication output increased across the study period. An exponential curve fitted in Microsoft Office Excel 2019 summarized the observed publication counts descriptively (*y* = 0.0124e^0.1052x^, R^2^ = 0.9425) (Figure 2). From 2005 to 2011, annual output ranged from 32 to 96 publications. Output increased from 123 publications in 2012 to 195 in 2020. During 2021–2025, output rose overall from 219 to 321 publications, despite a decrease from 248 in 2022 to 236 in 2023. Publications from 2025 accounted for 10.32% of the included literature.

**Figure 2:**
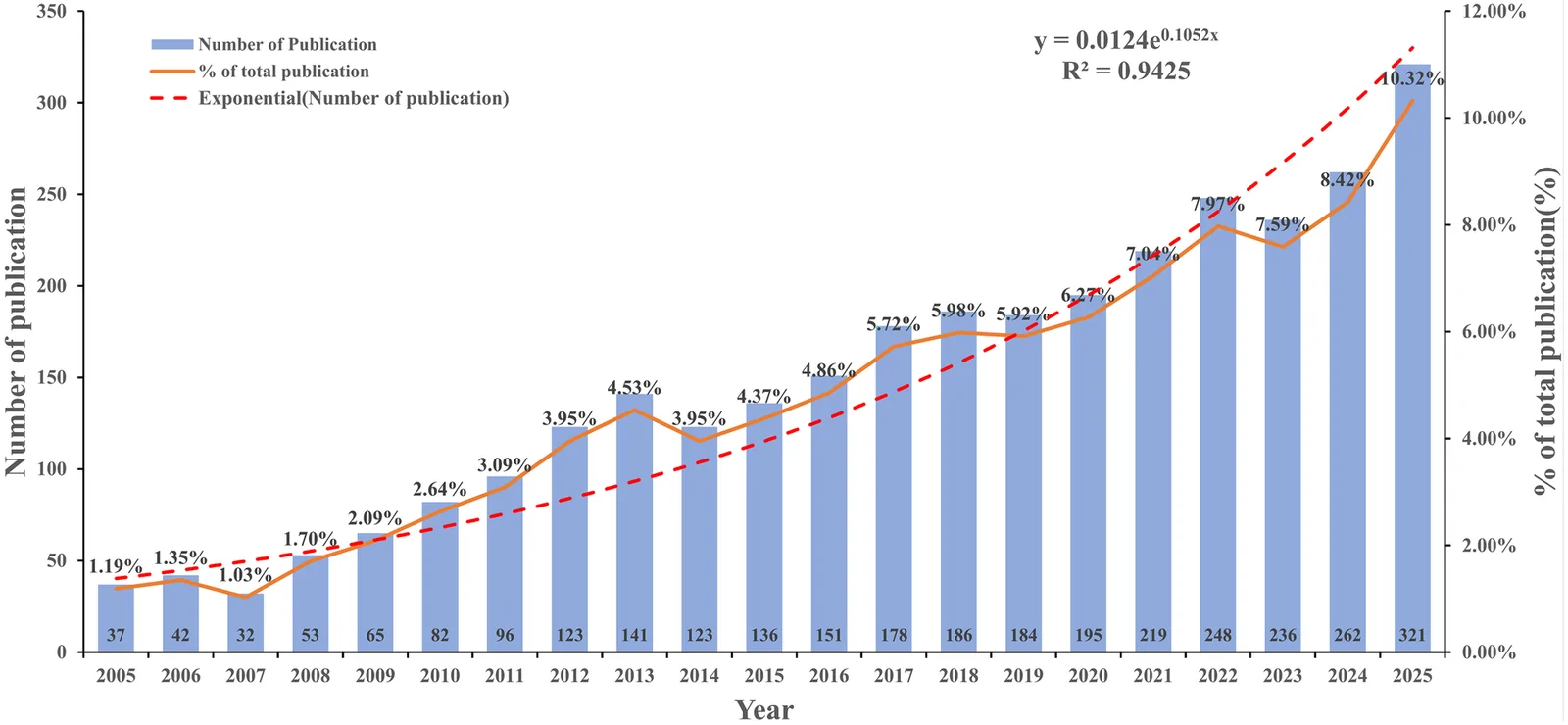
Annual publication output in knee osteoarthritis biomechanics research, 2005–2025.

### 3.2 Country/region, institution, author, and journal analyses

We compared publication output and total citations among the 10 leading countries or regions, institutions, authors, and journals. At the country level (Table 1), the United States ranked first in publication output (*n* = 900, 28.9%) and total citations (*n* = 40,710). China ranked second in publication output (*n* = 355, 11.4%) but fifth in total citations (*n* = 4,800). The collaboration network and multiple-country publication percentages (MCP%) are shown in Figures 3A,B. Among the 10 most productive countries, Australia (38.9%), the United Kingdom (36.9%), and Germany (28.0%) had relatively high MCP% values. They also occupied prominent positions in the international collaboration network. Institutional productivity analysis (Table 2) showed that the University of Melbourne (*n* = 140), Boston University (*n* = 100), and Stanford University (*n* = 83) were the three most productive institutions. Boston University had the highest total citation count (8,554), followed by Lund University (6,576) and the University of Melbourne (6,245).

**Table 1:** Publication output and citation metrics by country or region.

Rank	Top 10 most productive countries/regions						Most cited countries/regions		
	Country/region	Record	Percentage of corpus (%)	SCP	MCP	MCP%	Country/region	TC	AAC
1	USA	900	28.9	726	174	19.3	USA	40,710	45.2
2	CHINA	355	11.4	301	54	15.2	AUSTRALIA	10,568	43.3
3	CANADA	282	9.1	223	59	20.9	CANADA	9,681	34.3
4	AUSTRALIA	244	7.8	149	95	38.9	UNITED KINGDOM	8,870	52.8
5	JAPAN	220	7.1	207	13	5.9	CHINA	4,800	13.5
6	UNITED KINGDOM	168	5.4	106	62	36.9	JAPAN	4,232	19.2
7	GERMANY	107	3.4	77	30	28.0	NETHERLANDS	3,578	47.1
8	ITALY	82	2.6	61	21	25.6	SWEDEN	3,416	122
9	NETHERLANDS	76	2.4	57	19	25	GERMANY	2,699	25.2
10	KOREA	69	2.2	61	8	11.6	ITALY	2,016	24.6

TC, total citations; AAC, average citations per article; SCP, single-country publications; MCP, multiple-country publications; MCP%, percentage of multiple-country publications.

**Figure 3:**
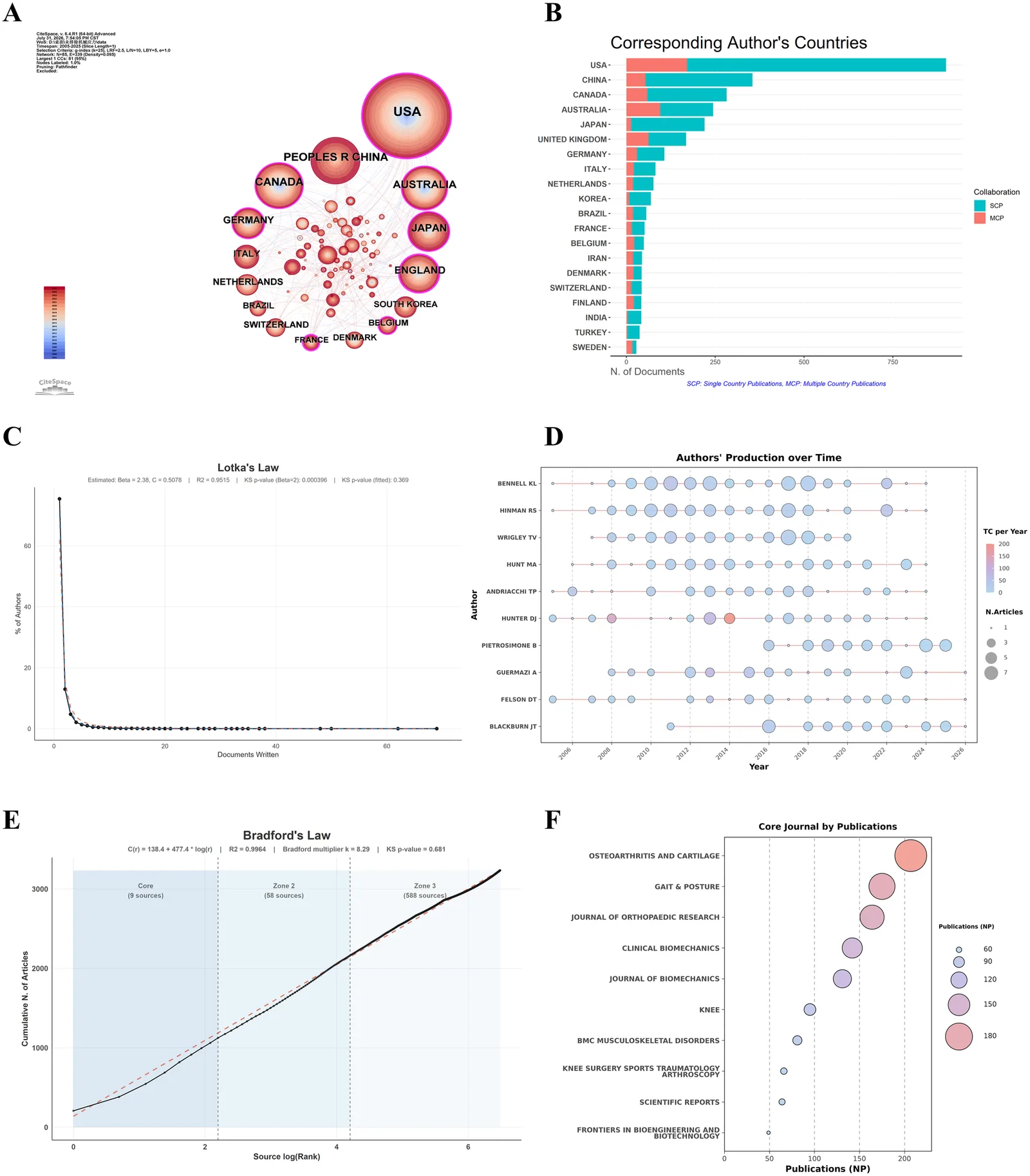
Geographic, author, and journal characteristics of knee osteoarthritis biomechanics research. **(A)** Country or region collaboration network generated with CiteSpace. **(B)** Numbers of single-country publications (SCPs) and multiple-country publications (MCPs) by corresponding author's country or region. **(C)** Observed author-productivity distribution and Lotka-type model fit. **(D)** Annual publication activity of the leading authors. **(E)** Bradford's law distribution. **(F)** Leading journals by publication output.

**Table 2:** Top 10 most productive and most cited institutions.

Rank	Top 10 most productive institutions			Most cited institutions			
	Name	Record	Country/region	Name	Country/region	TC	AAC
1	UNIV MELBOURNE	140	AUSTRALIA	BOSTON UNIV	USA	8,554	85.5
2	BOSTON UNIV	100	USA	LUND UNIV	SWEDEN	6,576	212.1
3	STANFORD UNIV	83	USA	UNIV MELBOURNE	AUSTRALIA	6,245	44.6
4	UNIV CALIF SAN FRANCISCO	82	USA	UNIV SYDNEY	AUSTRALIA	6,175	108.3
5	UNIV CALGARY	62	CANADA	UNIV OXFORD	UNITED KINGDOM	5,572	214.3
6	UNIV SYDNEY	57	AUSTRALIA	UNIV CALIF SAN FRANCISCO	USA	4,226	51.5
7	DALHOUSIE UNIV	57	CANADA	STANFORD UNIV	USA	3,981	48.0
8	UNIV BRITISH COLUMBIA	56	CANADA	DALHOUSIE UNIV	CANADA	3,516	61.7
9	UNIV DELAWARE	56	USA	UNIV SOUTHERN DENMARK	DENMARK	3,379	153.6
10	MONASH UNIV	55	AUSTRALIA	DUKE UNIV	USA	3,210	60.6

TC, total citations; AAC, average citations per article.

Author productivity followed a fitted Lotka-type distribution rather than the fixed classical *β* = 2 form. The fitted exponent was *β* = 2.38 (R^2^ = 0.9515), and the fitted distribution was not rejected by the Kolmogorov–Smirnov test (*p* = 0.369). By contrast, the fixed *β* = 2 model was rejected (*p* = 0.000396; Figure 3C). Approximately three-quarters of authors contributed only one publication, whereas a small proportion published multiple articles, producing a long-tail distribution. BENNELL KL (*n* = 69), HINMAN RS (*n* = 62), and WRIGLEY TV (*n* = 50), all affiliated with the University of Melbourne, ranked among the leading contributors by publication output and total citations (Table 3). The author timeline showed sustained activity by these three authors over an extended period (Figure 3D). It also showed increasing recent publication activity by other researchers, including PIETROSIMONE B and BLACKBURN JT from the University of North Carolina. This pattern indicates increasing diversity among active contributors to the field.

**Table 3:** Publication output and citation metrics for the 10 most productive authors.

Rank	Top 10 most productive authors					
	Name	Country/region	Institution	Record	TC	AAC
1	BENNELL KL	AUSTRALIA	UNIV MELBOURNE	69	2,594	37.6
2	HINMAN RS	AUSTRALIA	UNIV MELBOURNE	62	2,468	39.8
3	WRIGLEY TV	AUSTRALIA	UNIV MELBOURNE	50	1,915	38.3
4	HUNT MA	CANADA	UNIV BRITISH COLUMBIA	48	2,241	46.7
5	ANDRIACCHI TP	USA	STANFORD UNIV	38	2,543	66.9
6	HUNTER DJ	AUSTRALIA	UNIV SYDNEY	38	1,572	41.4
7	PIETROSIMONE B	USA	UNIV NORTH CAROLINA	37	665	18.0
8	GUERMAZI A	USA	BOSTON UNIV	35	2,600	74.3
9	FELSON DT	USA	BOSTON UNIV	34	1,531	45.0
10	BLACKBURN JT	USA	UNIV NORTH CAROLINA	33	644	19.5

TC, total citations; AAC, average citations per article.

We first identified core journals using Bradford's law. As shown in Figure 3E, nine journals formed the core, whereas Zone 2 and Zone 3 contained 58 and 588 journals, respectively. Regarding publication output (Figure 3F), Osteoarthritis and Cartilage ranked first (*n* = 207), followed by Gait & Posture (*n* = 175), Journal of Orthopaedic Research (*n* = 164), Clinical Biomechanics (*n* = 142), and Journal of Biomechanics (*n* = 131). In terms of total citations (Table 4), Osteoarthritis and Cartilage ranked first with 15,525 citations. It was followed by Journal of Orthopaedic Research (5,118) and Arthritis and Rheumatism (4,863). Thus, Osteoarthritis and Cartilage was both the most productive and the most frequently cited journal in this corpus.

**Table 4:** Publication output and citation metrics by journal.

Rank	Top 10 most productive journals				Most cited journals			
	Journal	Record	Percentage of corpus (%)	IF (2024)	Journal	TC	AJC	IF (2024)
1	OSTEOARTHRITIS AND CARTILAGE	207	6.7	9	OSTEOARTHRITIS AND CARTILAGE	15,525	75	9
2	GAIT & POSTURE	175	5.6	2.4	JOURNAL OF ORTHOPAEDIC RESEARCH	5,118	31.2	2.3
3	JOURNAL OF ORTHOPAEDIC RESEARCH	164	5.3	2.3	ARTHRITIS & RHEUMATOLOGY	4,863	127.0	10.9
4	CLINICAL BIOMECHANICS	142	4.6	1.4	GAIT & POSTURE	4,090	23.4	2.4
5	JOURNAL OF BIOMECHANICS	131	4.2	2.4	AMERICAN JOURNAL OF SPORTS MEDICINE	3,766	88.7	4.5
6	KNEE	95	3.1	2	JOURNAL OF BIOMECHANICS	3,442	26.3	2.4
7	BMC MUSCULOSKELETAL DISORDERS	81	2.6	2.4	CLINICAL BIOMECHANICS	3,226	22.7	1.4
8	KNEE SURGERY SPORTS TRAUMATOLOGY ARTHROSCOPY	66	2.1	5	NATURE REVIEWS RHEUMATOLOGY	2,855	190	32.7
9	SCIENTIFIC REPORTS	64	2.1	3.9	KNEE SURGERY SPORTS TRAUMATOLOGY ARTHROSCOPY	2,299	34.8	5
10	FRONTIERS IN BIOENGINEERING AND BIOTECHNOLOGY	49	1.6	4.8	KNEE	2,049	21.6	2

IF (2024), 2024 journal impact factor; TC, total citations; AJC, average citations per journal article.

### 3.3 Bibliometric analysis of documents

The 10 most-cited documents in the corpus are summarized in Table 5. Co-citation and citation-burst analyses further characterized the cited intellectual base of KOA biomechanics and temporal changes in citation attention (Figures 4A,B). From 2006 to 2013, prominent burst references helped establish the field's biomechanical framework. Zhao et al. used direct *in vivo* measurements of joint forces to validate the association between the KAM and medial-compartment contact force (28). Astephen et al. reported distinct lower-extremity joint kinetics and neuromuscular activation across KOA severity levels (29). Their study had a citation-burst strength of 15.19 that persisted until 2013. Citation attention subsequently shifted toward associations between abnormal mechanical loading and structural knee progression. Bennell et al. examined dynamic medial knee loading and cartilage loss (30). This reference showed a citation burst from 2012 to 2016, with a strength of 27.20. The work identified KAM impulse, which incorporates loading magnitude and duration, as a potentially important indicator of cartilage degeneration. Chehab et al., whose reference showed a citation-burst strength of 28.88 from 2015 to 2019, linked the KAM and knee flexion moment to cartilage changes over a 5-year period (31). This work extended the research framework beyond coronal-plane loading. During the same period, the Osteoarthritis Research Society International (OARSI) guidelines for nonsurgical KOA management by McAlindon et al. had a citation-burst strength of 18.75 from 2015 to 2019 (32). This burst indicated concentrated attention to clinical management, including exercise, weight management, bracing, and individualized conservative interventions. The guidelines emphasized tailoring treatment to individual patient characteristics. After 2017, citation bursts increasingly involved early-onset and post-traumatic KOA after anterior cruciate ligament (ACL) injury and reconstruction. Wellsandt et al. examined reduced knee loading after ACL injury and the development of early osteoarthritis; their study showed a citation burst from 2017 to 2021 (33). Pfeiffer et al. showed a sustained citation burst from 2020 through 2025, the end of the analysis period (34). Their integration of gait biomechanics with T1p magnetic resonance imaging (MRI) exemplified recent work combining mechanical assessment with quantitative cartilage imaging. Overall, the citation patterns suggest that the cited base broadened beyond external joint moments in established KOA. It increasingly included high-risk populations, early cartilage alterations, and post-traumatic disease. Studies by Zhao, Astephen, Bennell, Chehab, and McAlindon had both high co-citation frequencies and prominent citation bursts. They therefore represented prominent references in the cited intellectual base of KOA biomechanics.

**Table 5:** Top 10 most-cited documents.

Rank	Title	First author	Corresponding author	PY	Journal
1	Baseline knee adduction and flexion moments during walking are both associated with 5 year cartilage changes in patients with medial knee osteoarthritis	CHEHAB EF	CHEHAB EF	2014	OSTEOARTHRITIS AND CARTILAGE
2	Higher dynamic medial knee load predicts greater cartilage loss over 12 months in medial knee osteoarthritis	BENNELL KL	BENNELL KL	2011	ANNALS OF THE RHEUMATIC DISEASES
3	Osteoarthritis	HUNTER DJ	HUNTER DJ	2019	LANCET
4	OARSI guidelines for the non-surgical management of knee, hip, and polyarticular osteoarthritis	BANNURU RR	BANNURU RR	2019	OSTEOARTHRITIS AND CARTILAGE
5	External knee adduction and flexion moments during gait and medial tibiofemoral disease progression in knee osteoarthritis	CHANG AH	CHANG AH	2015	OSTEOARTHRITIS AND CARTILAGE
6	OARSI guidelines for the non-surgical management of knee osteoarthritis	MCALINDON TE	MCALINDON TE	2014	OSTEOARTHRITIS AND CARTILAGE
7	2019 American College of Rheumatology/Arthritis Foundation guideline for the management of osteoarthritis of the hand, hip, and knee	KOLASINSKI SL	KOLASINSKI SL	2020	ARTHRITIS CARE & RESEARCH
8	Global, regional prevalence, incidence and risk factors of knee osteoarthritis in population-based studies	CUI A	LU H	2020	ECLINICALMEDICINE
9	Correlation between the knee adduction torque and medial contact force for a variety of gait patterns	ZHAO D	FREGLY BJ	2007	JOURNAL OF ORTHOPAEDIC RESEARCH
10	Gait and neuromuscular pattern changes are associated with differences in knee osteoarthritis severity levels	ASTEPHEN JL	ASTEPHEN JL	2008	JOURNAL OF BIOMECHANICS

PY, publication year.

**Figure 4:**
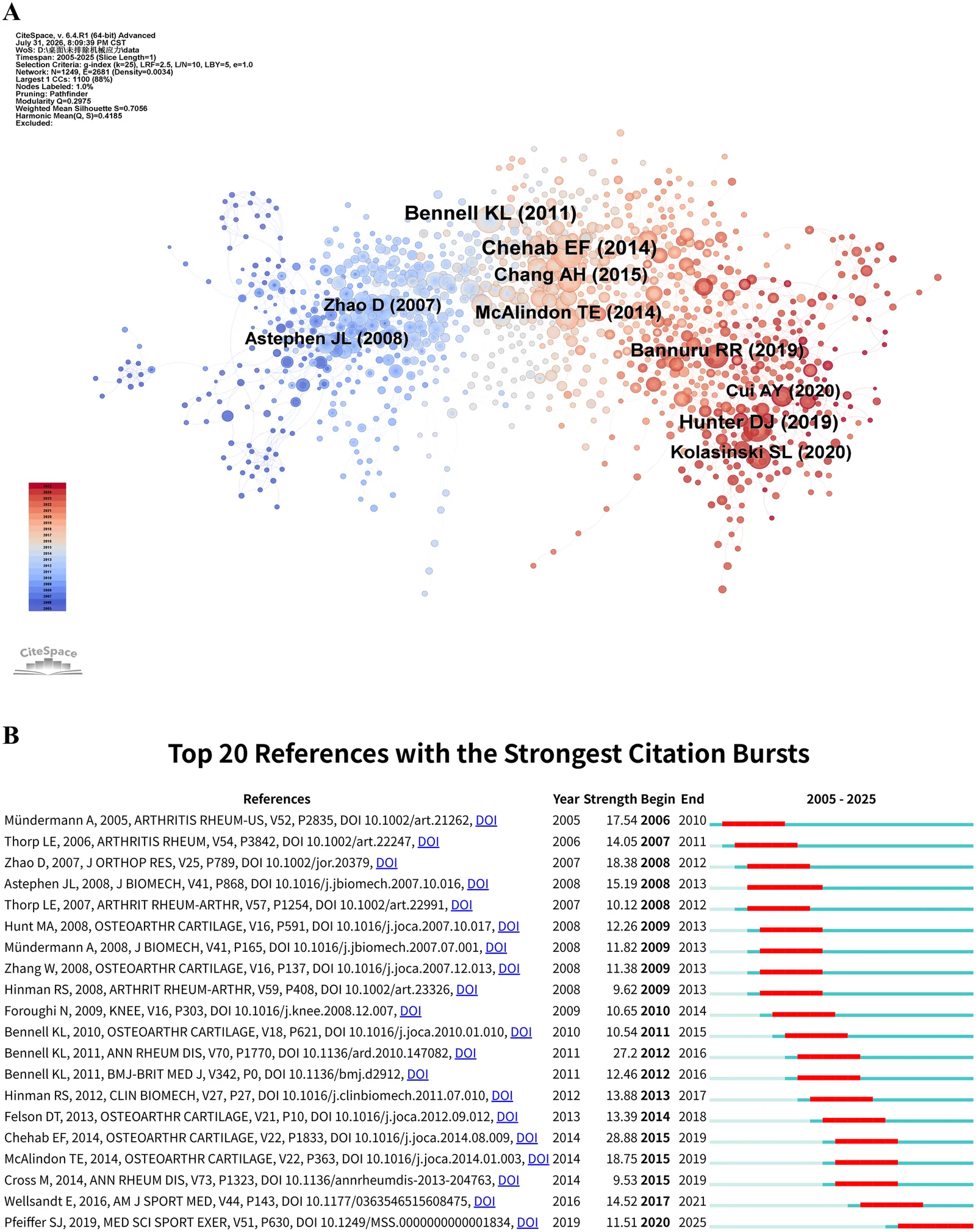
Cited-reference co-citation structure and citation bursts in knee osteoarthritis biomechanics research. **(A)** Cited-reference co-citation network generated with CiteSpace. **(B)** The 20 references with the strongest citation bursts.

### 3.4 Keyword cluster analysis

We used VOSviewer to analyze keyword co-occurrence and characterize the main themes in KOA biomechanics (Figure 5A). In the network, node size represented keyword frequency, and line thickness represented pairwise co-occurrence strength. Node colors denoted thematic clusters. The five color-coded clusters were interpreted as osteochondral-unit mechanobiology (red); gait biomechanics and neuromuscular control (green); joint-load modulation and conservative rehabilitation (blue); lower-limb alignment correction and knee reconstruction (yellow); and meniscal and ligament contact mechanics (purple).

**Figure 5:**
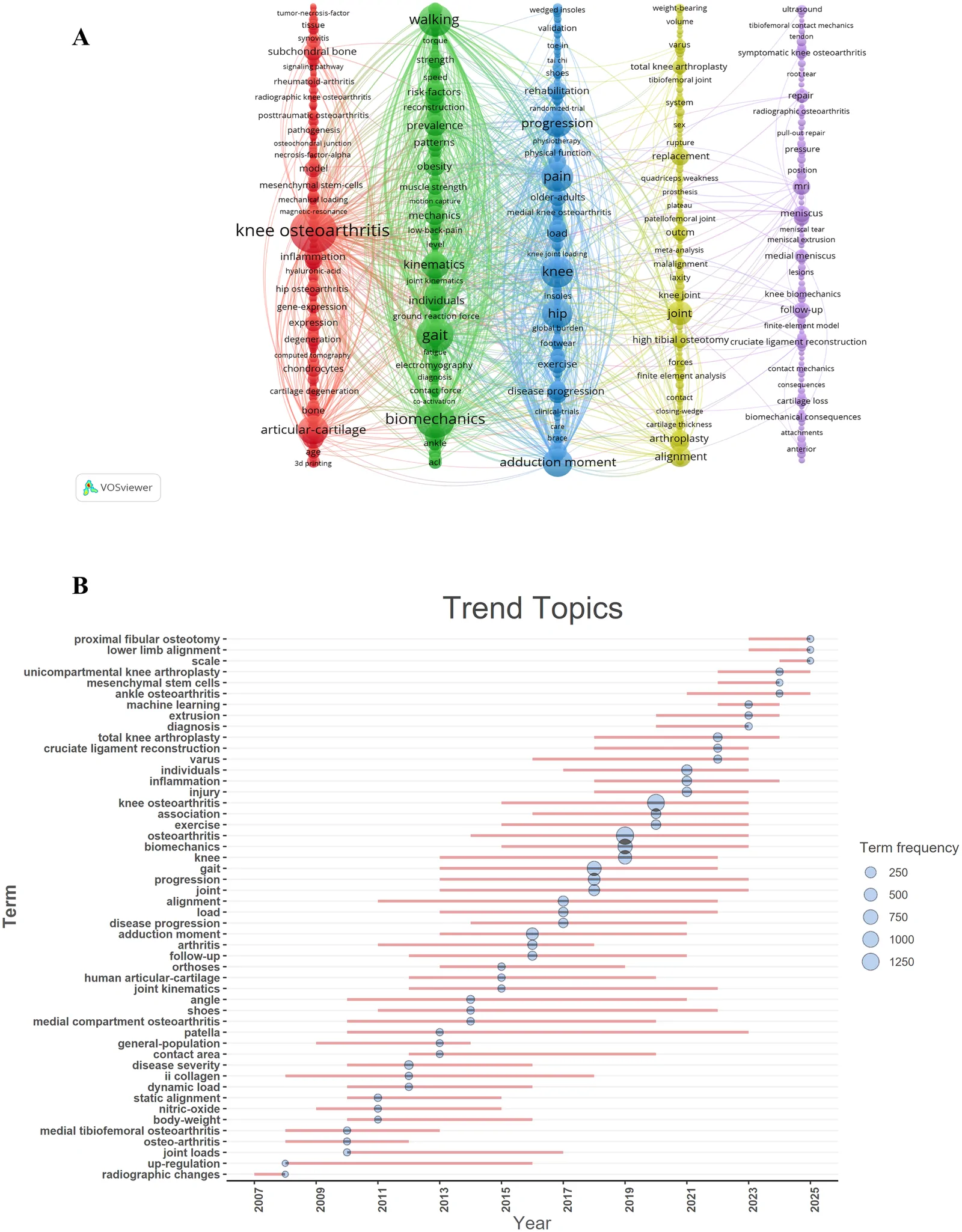
Author-keyword structure and temporal distribution in knee osteoarthritis biomechanics research. **(A)** Author-keyword co-occurrence network generated with VOSviewer using full counting and association-strength normalization. **(B)** Temporal distribution of selected keywords.

### 3.5 Keyword timeline analysis

Keyword timeline analysis characterized the temporal distribution of research terms in KOA biomechanics (Figure 5B). Terms concerning joint-preserving treatment, lower-limb alignment correction, regenerative repair, and machine-learning-assisted diagnosis were concentrated mainly in the latter half of the study period. Proximal fibular osteotomy, lower-limb alignment, and unicompartmental knee arthroplasty appeared predominantly after 2022. Mesenchymal stem cells also had a later temporal distribution, alongside recent work on cartilage regeneration and biological repair. Machine learning and diagnosis were similarly concentrated in the later period, including work on artificial intelligence-assisted diagnosis of early KOA. Overall, terms related to joint-preserving interventions, regenerative medicine, structural imaging, and artificial intelligence were concentrated in the later years of the study period.

### 3.6 Structure and evolution of research themes identified by machine learning

Four candidate models (*K* = 8, 9, 10, and 19) met the eligibility criteria. Among these, *K* = 19 had the highest mean NPMI (0.4225) and was the only model within the specified coherence tolerance of 0.02. Its initialization and subsample P10 similarities were 0.7744 and 0.7699, respectively, and increasing K from 19 to 20 reduced normalized reconstruction error by 0.1305%. Although *K* = 20 had a slightly higher mean NPMI (0.4283), it failed the initialization reproducibility criterion (P10 = 0.2688). *K* = 19 was therefore selected under the reported criteria (Figure 6A; Supplementary Tables S4–S6). Fourteen of the 19 topics met both topic-level reproducibility thresholds. Five topics fell below at least one threshold: T06 (initialization/subsample P10 = 0.91/0.17), T10 (0.18/0.17), T14 (0.16/0.15), T15 (0.85/0.12), and T18 (0.20/0.17) (Figure 6B). Accordingly, temporal patterns for these five topics were considered exploratory and were not used as standalone evidence of emerging or declining research priorities.

**Figure 6:**
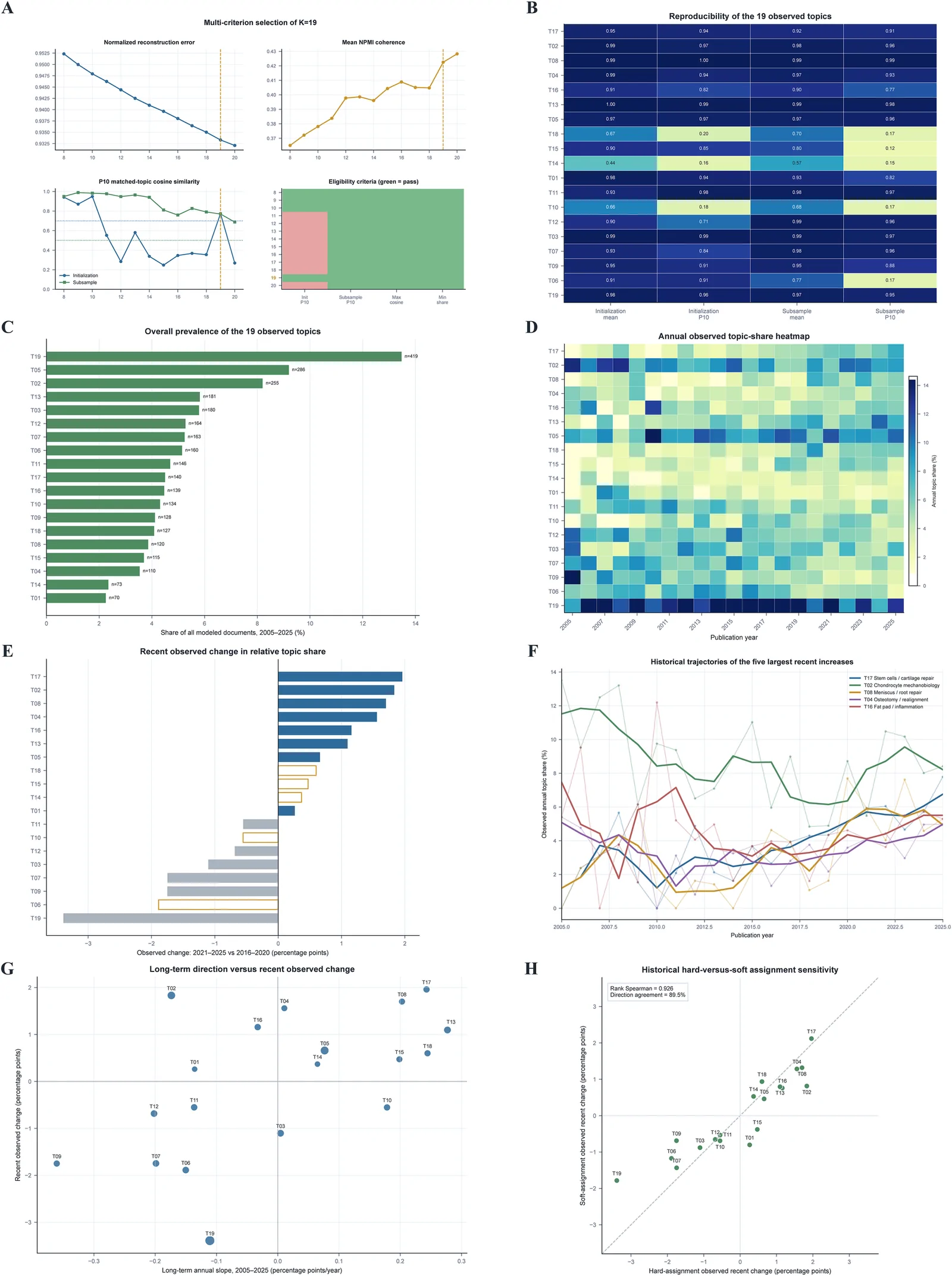
Selection, reproducibility, prevalence, and temporal distribution of topics identified by non-negative matrix factorization (NMF). **(A)** Candidate topic numbers (*K* = 8–20) were evaluated using the prespecified model-selection metrics; *K* = 19 was selected. **(B)** Reproducibility of the 19 topics across 30 full-corpus random-initialization refits and 50 year-stratified 80% subsample refits. **(C)** Overall prevalence of the 19 topics based on each document's highest-weight topic; labels show the number of assigned publications. **(D)** Annual topic-share heatmap based on highest-weight, or hard, topic assignment. **(E)** Difference in mean annual topic share between 2021–2025 and 2016–2020, expressed in percentage points. **(F)** Annual observed shares and smoothed trajectories for the five topics with the largest recent increases in mean annual share. **(G)** Comparison of the long-term linear slope for 2005–2025 with the recent change defined in panel E. **(H)** Sensitivity analysis comparing recent changes estimated using hard and normalized soft topic assignments.

The 19 topics delineated a multidimensional knowledge structure in KOA biomechanics (Table 6, Figure 6C). Gait and lower-limb kinematics (T19) accounted for the largest share of the literature. Exercise rehabilitation, pain, and physical function (T05) and chondrocyte mechanotransduction and cartilage matrix biology (T02) followed. These findings identify movement, rehabilitation, physical function, and cartilage mechanobiology as major directions in the corpus. The annual topic-share heatmap described changes in relative topic representation from 2005 to 2025 (Figure 6D). Gait and lower-limb kinematics (T19) remained prominent across most years, although its relative share was lower recently. Exercise rehabilitation, pain, and physical function (T05) also maintained a relatively high share throughout the study period. Chondrocyte mechanotransduction and cartilage matrix biology (T02) was comparatively prominent early, declined, and increased modestly in recent years. Stem cells and regenerative cartilage repair (T17) and Meniscal tears, extrusion, and root repair (T08) had larger relative shares in the middle and later periods. Finite-element modeling of joint stress and strain (T18) showed a similar pattern, although its low reproducibility limits confidence in this temporal interpretation. Conversely, body mass, obesity, and osteoarthritis risk (T09), footwear orthoses and lateral-wedge insoles (T06), and quantitative cartilage MRI and mechanical properties (T07) were more prominent in the early or middle periods. Their relative shares were smaller in recent years.

**Table 6:** Researcher-interpreted labels and representative terms for the 19 NMF-derived topics.

Topic ID	Researcher-interpreted topic	Representative high-weight terms
T01	Knee adduction moment and medial knee loading	Adduction; moment; adduction moment; kam; gait
T02	Chondrocyte mechanotransduction and cartilage matrix biology	Expression; chondrocytes; cartilage; chondrocyte; matrix
T03	Anterior cruciate ligament injury and reconstruction	acl; cruciate ligament; cruciate; ligament; anterior cruciate
T04	High tibial osteotomy and lower-limb realignment	Osteotomy; tibial; tibial osteotomy; high tibial; hto
T05	Exercise rehabilitation, pain, and physical function	Pain; exercise; function; physical; therapy
T06	Footwear orthoses and lateral-wedge insoles	Insoles; lateral wedge; wedge; insole; foot
T07	Quantitative cartilage MRI and mechanical properties	Cartilage; mri; imaging; relaxation; magnetic
T08	Meniscal tears, extrusion, and root repair	Meniscus; meniscal; extrusion; root; tears
T09	Body mass, obesity, and osteoarthritis risk	bmi; age; risk; index; mass
T10	Tibiofemoral contact forces and musculoskeletal modeling	Contact; forces; contact forces; force; contact force
T11	Muscle activation strength and neuromuscular control	Muscle; activation; muscle activation; quadriceps; strength
T12	Subchondral bone remodeling and microarchitecture	Bone; subchondral; subchondral bone; trabecular; cartilage
T13	Knee arthroplasty biomechanics and outcomes	Arthroplasty; tka; total arthroplasty; total; replacement
T14	Lower-limb amputation prosthetic gait and intact-limb loading	Limb; amputation; prosthetic; transtibial; lower limb
T15	ACL reconstruction and ground-reaction loading	aclr; ground reaction; reaction; ground; reaction force
T16	Adiposity, infrapatellar fat pad, and inflammation	Obesity; fat; inflammation; adipose; metabolic
T17	Stem cells and regenerative cartilage repair	Stem; cells; mesenchymal; stem cells; mesenchymal stem
T18	Finite-element modeling of joint stress and strain	Element; finite; finite element; element modeling; modeling
T19	Gait and lower-limb kinematics	Gait; hip; motion; kinematics; healthy

Topic identifiers are arbitrary model indices and do not indicate topic importance or ranking. Representative terms are listed according to their weights in the corresponding NMF component.

Relative to 2016–2020, the topics with the largest increases in mean annual share in 2021–2025 were stem cells and regenerative cartilage repair (T17), chondrocyte mechanotransduction and cartilage matrix biology (T02), and meniscal tears, extrusion, and root repair (T08). High tibial osteotomy and lower-limb realignment (T04) and adiposity, infrapatellar fat pad, and inflammation (T16) also had higher mean annual shares (Figure 6E). These changes describe a redistribution toward regenerative repair, cellular mechanobiology, local inflammatory microenvironments, and joint-preserving treatments. Over the same period, relative shares decreased for gait and lower-limb kinematics (T19), footwear orthoses and lateral-wedge insoles (T06), and body mass, obesity, and osteoarthritis risk (T09). The decrease in T06 was considered tentative because of its low subsample reproducibility. Quantitative cartilage MRI and mechanical properties (T07) and anterior cruciate ligament injury and reconstruction (T03) also had lower mean annual shares. Annual topic shares are compositional and sum to 100%. An increase in one necessarily reduces the combined share of the others. These changes reflect relative redistribution within an expanding corpus and do not by themselves establish absolute growth or decline in topic-specific publication counts. T19 remained the largest overall topic despite its lower recent share. The topics with the largest recent increases had variable rather than uniformly sustained or monotonic annual trajectories (Figure 6F). Recent differences should therefore be interpreted as period-specific, descriptive redistribution within the corpus. Stem cells and regenerative cartilage repair (T17), meniscal tears, extrusion, and root repair (T08), high tibial osteotomy and lower-limb realignment (T04), and adiposity, infrapatellar fat pad, and inflammation (T16) each had greater recent representation. Long-term slopes and recent changes were concordant for most topics, although some recent rebounds or declines differed from the longer-term trajectories (Figure 6G). For example, chondrocyte mechanotransduction and cartilage matrix biology (T02) showed a recent increase in mean annual share despite a negative full-period slope. Recent changes in topic share were strongly correlated between hard and normalized soft assignments (Spearman's *p* = 0.926), with concordant directions for 17 of 19 topics. The exceptions were T01 (knee adduction moment and medial knee loading) and T15 (ACL reconstruction and ground-reaction loading), both of which increased under hard assignment but decreased under soft assignment (Figure 6H). The recent direction of change for these two topics was therefore considered assignment-dependent. The WoSCC-restricted analysis showed high alignment for 18 of 19 topics (cosine similarity, 0.884–0.997), with concordant recent-change directions for 17 of these 18 pairs. T14 showed weak alignment, and T15 reversed direction, indicating broad consistency alongside topic-specific sensitivity to database restriction (Supplementary Table S7).

## 4 Discussion

### 4.1 General information

This study combined bibliometric mapping with NMF to characterize the development and thematic structure of 3,110 English-language publications on KOA biomechanics from 2005 to 2025. Publication output increased over the study period, while the integrated analyses identified established research domains and changes in their relative representation.

NMF complemented keyword mapping by distinguishing related themes and quantifying their temporal trajectories. Chondrocyte mechanotransduction (T02) showed a recent increase in relative share despite a negative full-period slope, whereas stem cells and regenerative cartilage repair (T17) showed both a positive full-period slope and a recent increase (Figures 6E–G). Gait and lower-limb kinematics (T19) remained the largest overall topic, although its mean annual share declined from 2016 to 2020 to 2021–2025 (Figures 6C–E). These findings distinguish recent rebounds from longer-term increases and overall prominence from recent redistribution. Such document-level comparisons were not directly represented by the keyword network or keyword-year summaries used in this study (Figure 5).

Research output was geographically concentrated. The United States led in publication output and total citations, while China ranked second in output but fifth in citations. Australia, the United Kingdom, and Germany had relatively high MCP% values, indicating comparatively extensive international collaboration (Table 1; Figures 3A,B). The University of Melbourne and its affiliated authors were prominent contributors (Tables 2, 3; Figure 3D). Broader multicenter collaboration may help assess whether findings generalize across populations and clinical settings. The leading journals, including Osteoarthritis and Cartilage, Gait & Posture, and Journal of Orthopaedic Research, reflect the field's connections with osteoarthritis research, movement science, and orthopaedic biomechanics (Table 4; Figures 3E,F). The country–topic analysis revealed both shared and distinct thematic emphases within national publication profiles (Supplementary Table S8). Gait and lower-limb kinematics (T19) ranked first in the United States, Canada, Japan, the United Kingdom, and Germany, suggesting a shared emphasis on movement analysis. Other leading topics included chondrocyte mechanotransduction and cartilage matrix biology (T02) in China, exercise rehabilitation (T05) in Australia, adiposity, infrapatellar fat pad, and inflammation (T16) in Italy, and high tibial osteotomy and lower-limb realignment (T04) in Korea. These patterns may partly reflect sustained contributions from groups with different methodological and clinical interests. For example, Australia's rehabilitation emphasis is consistent with the prominence of Melbourne-based researchers in our institutional and author analyses (Tables 2, 3). Although these possible explanations were not directly tested, the profiles suggest opportunities for collaboration connecting tissue-level mechanisms, movement analysis, and clinical interventions.

### 4.2 Research themes and their evolution

#### 4.2.1 Gait and joint loading as core research themes

Gait and lower-limb kinematics (T19) accounted for the largest overall topic share, while the keyword network identified clusters concerning gait, neuromuscular control, and load modulation (Figures 5A, 6C,D). These findings highlight the prominence of movement-based assessment in this corpus. Several highly cited publications and references showing citation bursts link gait measurements with joint loading and subsequent cartilage changes (Table 5; Figure 4B). Using *in vivo* joint-force measurements, Zhao et al. examined the relationship between KAM and medial tibiofemoral contact force across different gait patterns (28). Subsequent longitudinal studies have linked torque to changes in cartilage; KAM may serve as a risk factor for a reduction in medial cartilage volume, and KAM has a greater impact on femoral cartilage, whereas KFM focuses on the tibia (30, 31). However, KAM remains only a surrogate measure of medial tibiofemoral contact force, rather than a direct measurement; simply reducing the KAM value does not necessarily result in a proportional reduction in medial contact force (35, 36). Combining peak KAM and KFM improved the estimation of peak medial compartment loading compared with using peak KAM alone (37). Together, these studies help explain the sustained attention to dynamic loading as a potential link between movement patterns and structural progression.

The sustained prominence of exercise rehabilitation, pain, and physical function (T05) places biomechanical assessment within a broader clinical context. The OARSI guidelines for nonsurgical management of KOA were identified among both highly cited publications and references showing citation bursts, reflecting the importance placed on biomechanical interventions, weight management, and personalized care (32). The ACL-related references showing citation bursts add a complementary perspective on the meaning of altered loading (Figure 4B). Lower KAM and medial compartment joint contact load in the early post-ACL injury and reconstruction period are associated with late-stage radiographic findings of osteoarthritis, and lower mechanical loads during walking may also be associated with reduced proteoglycan density (33, 34). Gait mechanics and patellofemoral contact forces measured three months after ACL reconstruction were associated with MRI-based measures of patellofemoral cartilage health at 24 months (38). These thematic and citation patterns highlight the role of gait and joint-loading research in connecting mechanical assessment with structural and functional outcomes. Their clinical interpretation should account for injury history and joint status, because the implications of altered loading may differ between established KOA and the early period after ACL reconstruction. T03 and T15 represent related perspectives on ACL injury and reconstruction, with T15 placing greater emphasis on ground-reaction loading; their thematic boundaries therefore overlap.

#### 4.2.2 Temporal redistribution of research attention

Temporal topic patterns suggest a redistribution of attention within an expanding literature. Because annual topic shares sum to 100%, increases in some topics necessarily reduce the combined share of others; a smaller share does not establish a decline in absolute publication output. Compared with 2016–2020, the 2021–2025 period showed greater representation of chondrocyte mechanotransduction (T02) and stem cells and regenerative cartilage repair (T17), but their trajectories differed (Figures 6E–G). T02 showed a recent rebound despite a negative full-period slope, whereas T17 had both a positive full-period slope and a recent increase. Together with the increased recent share of adiposity, infrapatellar fat pad, and inflammation (T16), these patterns suggest greater relative attention to cellular responses, repair, and the local tissue environment. These findings suggest a broadening of research emphases alongside the continued overall prominence of gait research.

The higher recent shares of meniscal tears, extrusion, and root repair (T08) and high tibial osteotomy and lower-limb realignment (T04) likewise suggest increased relative attention to joint preservation (Figures 6E–G). In an Osteoarthritis Initiative cohort, greater medial meniscal extrusion was associated with structural progression and combined structural and symptomatic progression (39). Selected clinical studies have reported reduced medial meniscal extrusion after posterior root repair, including repair combined with meniscal centralization (40, 41). A systematic review reported improvements in mean and peak contact pressures and patient-reported outcomes after medial meniscus posterior root repair, with greater improvement in Lysholm scores among patients undergoing concomitant high tibial osteotomy (HTO) (42). For alignment correction, adding HTO to nonsurgical management reduced two-year medial tibiofemoral cartilage-thickness loss and improved KOOS in patients with varus alignment and medial-compartment KOA (43). HTO can correct lower limb alignment, reduce synovial inflammation, and promote the polarization of macrophages from M1 to M2, thereby improving the intra-articular environment (44).

Taken together, these temporal patterns suggest a broadening of research attention within KOA biomechanics. Alongside the overall prominence of gait and joint-loading research, the greater recent representation of tissue responses, regenerative repair, and joint preservation highlights related questions about how mechanical conditions affect joint tissues and how these relationships can inform joint-preserving strategies.

### 4.3 Potential topics for future development

Recent topic patterns showed greater relative representation of chondrocyte mechanotransduction (T02), stem cells and regenerative cartilage repair (T17), and adiposity, infrapatellar fat pad, and inflammation (T16) (Figures 6E–G). These findings suggest opportunities to connect joint-level loading with tissue responses and repair through longitudinal studies combining mechanical, imaging, and clinical assessments. The greater recent representation of meniscal tears, extrusion, and root repair (T08) and high tibial osteotomy and lower-limb realignment (T04) also highlights joint preservation as a direction for further investigation. Future work could examine whether intervention-related mechanical changes accompany sustained structural preservation, pain relief, and functional improvement.

Multimodal approaches integrating computational modeling, medical imaging, wearable sensors, and machine learning offer a potential framework for addressing these questions. Subject-specific musculoskeletal and finite-element models (T10 and T18) could incorporate gait measurements (T19) and imaging-derived anatomy to estimate joint loading and local tissue stress and strain. Quantitative cartilage MRI (T07) could provide complementary tissue measurements for examining how these mechanical estimates relate to cartilage condition and subsequent changes. Özdemir and Topsakal reported a shift from automated radiographic grading toward multimodal, interpretable KOA progression prediction (45). This evolution complements the mechanobiology and repair themes identified in our corpus, suggesting opportunities to evaluate biomechanical features alongside imaging and clinical information in prediction models. Wearable sensors could support repeated movement assessments during daily activities. Future studies could test the added value of biomechanical information for early detection and progression-risk assessment, validate prediction models in independent cohorts, and prospectively evaluate their usefulness for personalized intervention.

## 5 Limitations

This study has several limitations. First, although records were retrieved from the Web of Science Core Collection and Scopus, studies indexed only in other databases may have been omitted. Differences in database coverage, indexing, citation counts, and metadata structure may also have introduced residual inconsistencies despite merging, deduplication, and standardization. Second, restricting the corpus to English-language articles and reviews may have introduced language and publication-type biases. Articles and reviews were analyzed together, and differences in their textual content and citation characteristics may have influenced the observed topic and citation patterns. Excluding conference proceedings may underrepresent computational and engineering developments, including modeling and gait-analysis methods, while excluding non-English publications may omit region-specific research priorities and rehabilitation practices. Broader eligibility criteria might increase the representation of these contributions and alter topic composition, relative shares, and temporal patterns; however, these effects were not assessed. The findings therefore characterize the included corpus and may not fully represent the global KOA biomechanics literature. Third, publication age, citation lag, journal visibility, self-citation, and database-specific citation practices affect bibliometric indicators. Recent publications had less time to accumulate citations. Citation counts should therefore not be interpreted as direct measures of methodological quality or clinical importance. Fourth, repeated refitting and sensitivity analyses comparing hard and soft assignment supported the overall robustness of the findings. Some topics may nevertheless overlap or have limited reproducibility. Finally, differences in available text metadata between WoSCC and Scopus may have influenced topic representation in the NMF analysis.

## 6 Conclusion

By integrating bibliometric mapping with NMF-based topic modeling, this study characterized the development and thematic distribution of KOA biomechanics research from 2005 to 2025. The field expanded substantially and remained centered on gait, lower-limb kinematics, exercise, rehabilitation, and joint-loading assessment. Recent literature showed greater relative representation of osteochondral and chondrocyte mechanobiology, inflammatory tissue microenvironments, meniscal extrusion and root repair, and osteotomy and alignment correction. Regenerative cartilage repair also had greater recent representation. Together, the citation, keyword, and NMF analyses describe a research landscape spanning whole-joint mechanics, tissue responses, and joint-preserving interventions targeting mechanical factors. Future work should prioritize longitudinal, externally validated multiscale models; standardized biomechanical and clinical outcomes; and direct tests of whether mechanical changes yield durable clinical or structural benefits.

## Data Availability

The datasets presented in this study can be found in online repositories. The names of the repository/repositories and accession number(s) can be found in the article/Supplementary Material.
