# Supplementary material for: Evolution of knee osteoarthritis biomechanics research, 2005–2025: bibliometric mapping and machine-learning-assisted topic modeling
Source: Front Med (Lausanne). 2026 Oct 26;13:1968099. doi: 10.3389/fmed.2026.1968099 (PMC13745276; doi:10.3389/fmed.2026.1968099)
Supplement: Supplementary file 1 [file Datasheet1.docx]

**Table S1: Database-specific search strategies.**

| **Database** | **Search strategy** |
| --- | --- |
| **WoSCC** | #1: TS=("knee osteoarthri*") OR TS=(KOA) OR TS=(knee OA) OR TS=("Osteoarthritis of the Knee") OR TS=("Osteoarthritis of knee")  #2: TS=("mechanical load") OR TS=("mechanical stress*") OR TS=("mechanical force") OR TS=("mechanical stimulation") OR TS=("mechanical compression") OR TS=("biomechanic*") OR TS=("joint loading") OR TS=("cartilage stress")  #1 AND #2 |
| **Scopus** | TITLE-ABS-KEY ("knee osteoarthri*" OR KOA OR "knee OA" OR "Osteoarthritis of the Knee" OR "Osteoarthritis of knee") AND TITLE-ABS-KEY ("mechanical load" OR "mechanical stress*" OR "mechanical force" OR "mechanical stimulation" OR "mechanical compression" OR biomechanic* OR "joint loading" OR "cartilage stress") |

Table S2: Text preprocessing and NMF model parameters

| **Component** | **Parameter** | **Setting used** |
| --- | --- | --- |
| Corpus | Documents / years | 3,110 / 2005–2025 |
| Corpus | Document types / language | Article and Review / English |
| Input | Text fields | Title, abstract, cleaned author keywords and cleaned Keywords Plus(WoSCC only) concatenated with spaces |
| Input | Feature unit | Word unigrams and bigrams; multi-word author keywords were not retained as indivisible features |
| TF-IDF | analyzer / lowercase | word / True |
| TF-IDF | ngram_range | (1, 2) |
| TF-IDF | min_df | 5 documents |
| TF-IDF | max_df | 0.85 of documents; terms occurring in more than 85% were excluded |
| TF-IDF | max_features / fitted vocabulary size | 6,000 / 6,000 |
| TF-IDF | stop_words | 400 unique terms: union of scikit-learn ENGLISH_STOP_WORDS (318), domain terms (36) and method/publication terms (49) |
| TF-IDF | sublinear_tf / norm | True / l2 |
| TF-IDF | use_idf / smooth_idf | True / True |
| TF-IDF | binary / strip_accents | False / None |
| TF-IDF | Custom tokenizer / preprocessor | None / None in the fitted vectorizer |
| NMF | Candidate n_components | 8–20 inclusive |
| NMF | Selected n_components | 19 |
| NMF | Baseline and final initialization | nndsvda |
| NMF | solver / beta_loss | cd (coordinate descent) / frobenius |
| NMF | max_iter / random_state | 1000 / 42 |
| Coherence | Top terms per topic | 10 |
| Reproducibility | Random-initialization refits per K | 30 |
| Reproducibility | Year-stratified subsample refits per K | 50; fraction 0.80; without replacement |

Table S3: Complete stop word list for NMF text preprocessing

| **Stop words** | **Stop words** | **Stop words** | **Stop words** | **Stop words** |
| --- | --- | --- | --- | --- |
| a | about | above | across | after |
| afterwards | again | against | aim | aims |
| all | almost | alone | along | already |
| also | although | always | am | among |
| amongst | amoungst | amount | an | analyses |
| analysis | and | another | any | anyhow |
| anyone | anything | anyway | anywhere | are |
| around | article | articles | as | assess |
| assessed | assessment | assessments | at | back |
| background | be | became | because | become |
| becomes | becoming | been | before | beforehand |
| behind | being | below | beside | besides |
| between | beyond | bias | bibliometric | bibliometrics |
| bill | both | bottom | but | by |
| call | can | cannot | cant | case |
| cases | clinical | co | cochrane | con |
| conclusion | conclusions | could | couldnt | cry |
| data | database | databases | de | describe |
| detail | disease | diseases | do | done |
| down | due | during | each | eg |
| eight | either | eleven | eligibility | eligible |
| else | elsewhere | empty | enough | etc |
| even | ever | every | everyone | everything |
| everywhere | evidence | evidencebased | except | excluded |
| exclusion | few | fifteen | fifty | fill |
| find | fire | first | five | for |
| former | formerly | forty | found | four |
| from | front | full | further | get |
| give | go | group | groups | had |
| has | hasnt | have | he | hence |
| her | here | hereafter | hereby | herein |
| hereupon | hers | herself | him | himself |
| his | how | however | hundred | i |
| ie | if | in | inc | included |
| inclusion | indeed | interest | into | is |
| it | its | itself | keep | knee |
| knees | koa | last | latter | latterly |
| least | less | literature | ltd | made |
| many | may | me | meanwhile | meta |
| metaanalyses | metaanalysis | method | methods | might |
| mill | mine | more | moreover | most |
| mostly | move | much | must | my |
| myself | name | namely | neither | never |
| nevertheless | next | nine | no | nobody |
| none | noone | nor | not | nothing |
| now | nowhere | objective | objectives | of |
| off | often | on | once | one |
| only | onto | or | osteoarthritis | osteoarthrosis |
| other | others | otherwise | our | ours |
| ourselves | out | over | own | paper |
| papers | part | patient | patients | per |
| perhaps | please | pooled | pooling | prisma |
| protocol | publication | publications | pubmed | purpose |
| put | randomised | randomized | rather | re |
| registration | reporting | result | results | review |
| reviews | same | searched | searches | searching |
| see | seem | seemed | seeming | seems |
| serious | several | she | should | show |
| side | since | sincere | six | sixty |
| so | some | somehow | someone | something |
| sometime | sometimes | somewhere | still | studies |
| study | subject | subjects | such | system |
| systematic | systematically | take | ten | than |
| that | the | their | them | themselves |
| then | thence | there | thereafter | thereby |
| therefore | therein | thereupon | these | they |
| thick | thin | third | this | those |
| though | three | through | throughout | thru |
| thus | to | together | too | top |
| toward | towards | trial | trials | twelve |
| twenty | two | un | under | until |
| up | upon | us | use | used |
| using | very | via | was | we |
| well | were | what | whatever | when |
| whence | whenever | where | whereafter | whereas |
| whereby | wherein | whereupon | wherever | whether |
| which | while | whither | who | whoever |
| whole | whom | whose | why | will |
| with | within | without | would | yet |
| you | your | yours | yourself | yourselves |

Table S4: NMF model selection across candidate topic numbers

| **K** | **Normalized reconstruction error** | **Mean NPMI** | **Maximum inter-topic cosine** | **Mean inter-topic cosine** | **Minimum prevalence (%)** | **Maximum prevalence (%)** | **Next-K error improvement (%)** | **Basic eligibility** | **Selected** |
| --- | --- | --- | --- | --- | --- | --- | --- | --- | --- |
| 8 | 0.9523 | 0.3650 | 0.3918 | 0.1828 | 7.66 | 18.42 | 0.2486 | Yes | No |
| 9 | 0.9500 | 0.3721 | 0.3890 | 0.1698 | 5.45 | 15.30 | 0.2155 | Yes | No |
| 10 | 0.9479 | 0.3781 | 0.3767 | 0.1620 | 5.15 | 14.62 | 0.1784 | Yes | No |
| 11 | 0.9462 | 0.3838 | 0.3628 | 0.1556 | 4.90 | 13.57 | 0.1950 | No | No |
| 12 | 0.9444 | 0.3978 | 0.3623 | 0.1474 | 4.65 | 13.24 | 0.1999 | No | No |
| 13 | 0.9425 | 0.3986 | 0.3621 | 0.1402 | 4.50 | 12.41 | 0.1618 | No | No |
| 14 | 0.9410 | 0.3961 | 0.3269 | 0.1350 | 4.36 | 11.94 | 0.1425 | No | No |
| 15 | 0.9396 | 0.4043 | 0.3266 | 0.1311 | 4.26 | 11.35 | 0.1693 | No | No |
| 16 | 0.9380 | 0.4089 | 0.3224 | 0.1271 | 4.11 | 10.64 | 0.1696 | No | No |
| 17 | 0.9365 | 0.4051 | 0.3212 | 0.1238 | 3.64 | 9.77 | 0.1533 | No | No |
| 18 | 0.9350 | 0.4048 | 0.3193 | 0.1172 | 3.51 | 8.24 | 0.1834 | No | No |
| **19** | **0.9333** | **0.4225** | **0.3179** | **0.1176** | **3.40** | **10.56** | **0.1305** | **Yes** | **Yes** |
| 20 | 0.9321 | 0.4283 | 0.3176 | 0.1132 | 3.29 | 8.83 | — | No | No |

Table S5: Reproducibility of candidate NMF models

| **K** | **Random initialization Mean** | **Random initialization Median** | **Random initialization P10** | **Random initialization Minimum** | **Document subsample Mean** | **Document subsample Median** | **Document subsample P10** | **Document subsample Minimum** |
| --- | --- | --- | --- | --- | --- | --- | --- | --- |
| 8 | 0.9374 | 0.9980 | 0.9418 | 0.2137 | 0.9598 | 0.9941 | 0.9493 | 0.1210 |
| 9 | 0.9426 | 0.9999 | 0.8706 | 0.1302 | 0.9862 | 0.9949 | 0.9892 | 0.1434 |
| 10 | 0.9590 | 1.0000 | 0.9492 | 0.0704 | 0.9768 | 0.9943 | 0.9827 | 0.1090 |
| 11 | 0.8785 | 0.9932 | 0.5521 | 0.0664 | 0.9680 | 0.9935 | 0.9772 | 0.0725 |
| 12 | 0.8551 | 0.9880 | 0.2840 | 0.0579 | 0.9521 | 0.9928 | 0.9479 | 0.0565 |
| 13 | 0.8869 | 0.9957 | 0.5800 | 0.0576 | 0.9597 | 0.9930 | 0.9645 | 0.0545 |
| 14 | 0.8565 | 0.9832 | 0.3373 | 0.0546 | 0.9485 | 0.9917 | 0.9407 | 0.0450 |
| 15 | 0.8302 | 0.9690 | 0.2475 | 0.0227 | 0.9086 | 0.9892 | 0.8110 | 0.0316 |
| 16 | 0.8600 | 0.9822 | 0.3458 | 0.0303 | 0.9058 | 0.9875 | 0.7593 | 0.0339 |
| 17 | 0.8813 | 0.9920 | 0.3678 | 0.0599 | 0.9149 | 0.9870 | 0.8271 | 0.0423 |
| 18 | 0.8627 | 0.9880 | 0.3546 | 0.0691 | 0.9004 | 0.9848 | 0.7908 | 0.0251 |
| **19** | **0.8971** | **0.9927** | **0.7744** | **0.0469** | **0.8954** | **0.9855** | **0.7699** | **0.0556** |
| 20 | 0.8753 | 0.9895 | 0.2688 | 0.0375 | 0.8824 | 0.9826 | 0.6877 | 0.0326 |

Table S6: Thresholds and sequential rules for selecting K

| **Step** | **Criterion** | **Rule used in the recorded analysis** | **Result for K=19** |
| --- | --- | --- | --- |
| 1 | Initialization reproducibility | P10 cosine similarity ≥ 0.70 | 0.774437; criterion met |
| 1 | Subsample reproducibility | P10 cosine similarity ≥ 0.50 | 0.769900; criterion met |
| 1 | Topic distinctiveness | Maximum inter-topic cosine similarity ≤ 0.95 | 0.317931; criterion met |
| 1 | Topic prevalence | Minimum soft-topic prevalence ≥ 0.005 (0.5%) | 0.034041 (3.4041%); criterion met |
| 2 | Coherence tolerance | Mean NPMI ≥ maximum NPMI among eligible K values minus 0.02 | Eligible maximum 0.422537; cutoff 0.402537; only K=19 retained |
| 3 | Model-fit plateau | Relative error improvement from K to K+1 ≤ 0.01 (1%) | 0.00130509 (0.130509%); criterion met |
| 4 | Final choice | Smallest K satisfying eligibility, coherence tolerance and the plateau condition | K=19; automatic selection; no user-specified K |
| 5 | Fallback if no plateau candidate | Select the smallest coherent eligible K | Not invoked |
| 5 | Fallback if no eligible model | Order by subsample P10, initialization P10 and NPMI descending, then K ascending | Not invoked |

Table S7: Topic alignment and temporal-trend comparison between the combined-corpus and WoSCC-restricted NMF models.

| **Topic ID** | **Topic label** | **Matched WoSCC topic** | **Cosine similarity** | **Main-model recent change (pp)** | **WoSCC-model recent change (pp)** | **Direction agreement** |
| --- | --- | --- | --- | --- | --- | --- |
| T01 | Knee adduction moment and medial knee loading | WS01 | 0.995 | +0.26 | +0.17 | Yes |
| T02 | Chondrocyte mechanotransduction and cartilage matrix biology | WS02 | 0.996 | +1.83 | +1.41 | Yes |
| T03 | Anterior cruciate ligament injury and reconstruction | WS03 | 0.997 | -1.10 | -1.78 | Yes |
| T04 | High tibial osteotomy and lower-limb realignment | WS15 | 0.994 | +1.56 | +1.95 | Yes |
| T05 | Exercise rehabilitation pain and physical function | WS05 | 0.975 | +0.66 | +1.61 | Yes |
| T06 | Footwear orthoses and lateral-wedge insoles | WS06 | 0.994 | -1.89 | -2.09 | Yes |
| T07 | Quantitative cartilage MRI and mechanical properties | WS04 | 0.996 | -1.75 | -1.49 | Yes |
| T08 | Meniscal tears extrusion and root repair | WS09 | 0.995 | +1.70 | +1.86 | Yes |
| T09 | Body mass obesity and osteoarthritis risk | WS10 | 0.973 | -1.75 | -0.67 | Yes |
| T10 | Tibiofemoral contact forces and musculoskeletal modeling | WS13 | 0.993 | -0.55 | -1.03 | Yes |
| T11 | Muscle activation strength and neuromuscular control | WS19 | 0.884 | -0.55 | -0.79 | Yes |
| T12 | Subchondral bone remodeling and microarchitecture | WS12 | 0.997 | -0.68 | -1.20 | Yes |
| T13 | Knee arthroplasty biomechanics and outcomes | WS07 | 0.989 | +1.10 | +1.17 | Yes |
| T14 | Lower-limb amputation prosthetic gait and intact-limb loading | WS11 | 0.177 | +0.37 | +0.97 | Not interpreted |
| T15 | ACL reconstruction and ground-reaction loading | WS14 | 0.989 | +0.47 | -0.88 | No |
| T16 | Adiposity infrapatellar fat pad and inflammation | WS16 | 0.977 | +1.16 | +0.38 | Yes |
| T17 | Stem cells and regenerative cartilage repair | WS17 | 0.991 | +1.96 | +2.08 | Yes |
| T18 | Finite-element modeling of joint stress and strain | WS08 | 0.996 | +0.60 | +0.71 | Yes |
| T19 | Gait and lower-limb kinematics | WS18 | 0.938 | -3.39 | -2.35 | Yes |

**Table S8: Distribution of the 19 NMF topics across the ten leading countries/regions in the included corpus, 2005–2025.**

| **Topic** | **USA (N = 900)** | **CHINA (N = 355)** | **CANADA (N = 282)** | **AUSTRALIA (N = 244)** | **JAPAN (N = 220)** | **UNITED KINGDOM (N = 168)** | **GERMANY (N = 107)** | **ITALY (N = 82)** | **NETHERLANDS (N = 76)** | **KOREA (N = 69)** |
| --- | --- | --- | --- | --- | --- | --- | --- | --- | --- | --- |
| T01 | 28 (3.1%) | 6 (1.7%) | 11 (3.9%) | 15 (6.1%) | 3 (1.4%) | 1 (0.6%) | 0 (0.0%) | 0 (0.0%) | 0 (0.0%) | 3 (4.3%) |
| T02 | 76 (8.4%) | 62 (17.5%) | 7 (2.5%) | 3 (1.2%) | 35 (15.9%) | 15 (8.9%) | 13 (12.1%) | 8 (9.8%) | 13 (17.1%) | 5 (7.2%) |
| T03 | 88 (9.8%) | 10 (2.8%) | 10 (3.5%) | 24 (9.8%) | 6 (2.7%) | 11 (6.5%) | 2 (1.9%) | 6 (7.3%) | 2 (2.6%) | 3 (4.3%) |
| T04 | 13 (1.4%) | 15 (4.2%) | 10 (3.5%) | 0 (0.0%) | 16 (7.3%) | 2 (1.2%) | 7 (6.5%) | 2 (2.4%) | 9 (11.8%) | 10 (14.5%) |
| T05 | 45 (5.0%) | 25 (7.0%) | 35 (12.4%) | 37 (15.2%) | 3 (1.4%) | 14 (8.3%) | 4 (3.7%) | 6 (7.3%) | 8 (10.5%) | 3 (4.3%) |
| T06 | 24 (2.7%) | 11 (3.1%) | 29 (10.3%) | 17 (7.0%) | 16 (7.3%) | 16 (9.5%) | 9 (8.4%) | 0 (0.0%) | 2 (2.6%) | 5 (7.2%) |
| T07 | 73 (8.1%) | 2 (0.6%) | 13 (4.6%) | 8 (3.3%) | 5 (2.3%) | 7 (4.2%) | 11 (10.3%) | 1 (1.2%) | 4 (5.3%) | 4 (5.8%) |
| T08 | 45 (5.0%) | 7 (2.0%) | 0 (0.0%) | 8 (3.3%) | 26 (11.8%) | 4 (2.4%) | 9 (8.4%) | 6 (7.3%) | 0 (0.0%) | 3 (4.3%) |
| T09 | 34 (3.8%) | 7 (2.0%) | 8 (2.8%) | 8 (3.3%) | 11 (5.0%) | 12 (7.1%) | 1 (0.9%) | 2 (2.4%) | 8 (10.5%) | 2 (2.9%) |
| T10 | 55 (6.1%) | 9 (2.5%) | 14 (5.0%) | 17 (7.0%) | 5 (2.3%) | 6 (3.6%) | 8 (7.5%) | 1 (1.2%) | 1 (1.3%) | 2 (2.9%) |
| T11 | 25 (2.8%) | 15 (4.2%) | 42 (14.9%) | 12 (4.9%) | 7 (3.2%) | 7 (4.2%) | 2 (1.9%) | 0 (0.0%) | 2 (2.6%) | 1 (1.4%) |
| T12 | 38 (4.2%) | 44 (12.4%) | 5 (1.8%) | 20 (8.2%) | 11 (5.0%) | 16 (9.5%) | 6 (5.6%) | 1 (1.2%) | 0 (0.0%) | 0 (0.0%) |
| T13 | 32 (3.6%) | 20 (5.6%) | 15 (5.3%) | 9 (3.7%) | 22 (10.0%) | 10 (6.0%) | 8 (7.5%) | 11 (13.4%) | 5 (6.6%) | 7 (10.1%) |
| T14 | 46 (5.1%) | 4 (1.1%) | 6 (2.1%) | 1 (0.4%) | 1 (0.5%) | 6 (3.6%) | 1 (0.9%) | 1 (1.2%) | 0 (0.0%) | 1 (1.4%) |
| T15 | 76 (8.4%) | 7 (2.0%) | 8 (2.8%) | 8 (3.3%) | 3 (1.4%) | 2 (1.2%) | 1 (0.9%) | 0 (0.0%) | 1 (1.3%) | 1 (1.4%) |
| T16 | 45 (5.0%) | 17 (4.8%) | 5 (1.8%) | 13 (5.3%) | 3 (1.4%) | 4 (2.4%) | 0 (0.0%) | 15 (18.3%) | 4 (5.3%) | 0 (0.0%) |
| T17 | 37 (4.1%) | 33 (9.3%) | 4 (1.4%) | 4 (1.6%) | 4 (1.8%) | 7 (4.2%) | 4 (3.7%) | 14 (17.1%) | 3 (3.9%) | 3 (4.3%) |
| T18 | 25 (2.8%) | 31 (8.7%) | 1 (0.4%) | 4 (1.6%) | 6 (2.7%) | 5 (3.0%) | 3 (2.8%) | 1 (1.2%) | 2 (2.6%) | 9 (13.0%) |
| T19 | 95 (10.6%) | 30 (8.5%) | 59 (20.9%) | 36 (14.8%) | 37 (16.8%) | 23 (13.7%) | 18 (16.8%) | 7 (8.5%) | 12 (15.8%) | 7 (10.1%) |
